# Supplementary material for: Substitution of H Atoms in Unsaturated (Vinyl-Type) Carbocations by Cl or O Atoms
Source: Int J Mol Sci. 2023 Jun 27;24(13):10734. doi: 10.3390/ijms241310734 (PMC10341854; doi:10.3390/ijms241310734)
Supplement: Supplementary file 1 [file ijms-24-10734-s001.zip › ijms-2411984-supplementary.pdf]

SUPPORTING INFORMATION

Substitution of H atoms in unsaturated (vinyl-type) carbocations by Cl or O atoms

Evgenii S. Stoyanov [*], *Irina Yu. Bagryanskaya and Irina V. Stoyanova*

N.N. Vorozhtsov Institute of Organic Chemistry, Siberian Branch of Russian Academy of Sciences, 630090 Novosibirsk, Russia

* Corresponding author. E-mail address: evgenii@nioch.nsc.ru

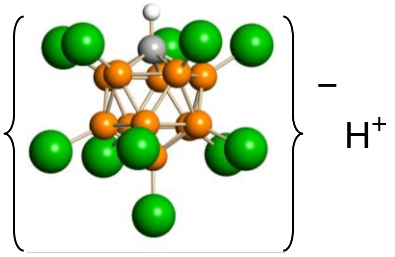

Figure S1. The super acid with icosahedral carborane anion $CHB_{11}Cl_{11}^{-}$, which was used for obtaining the carbocation salts

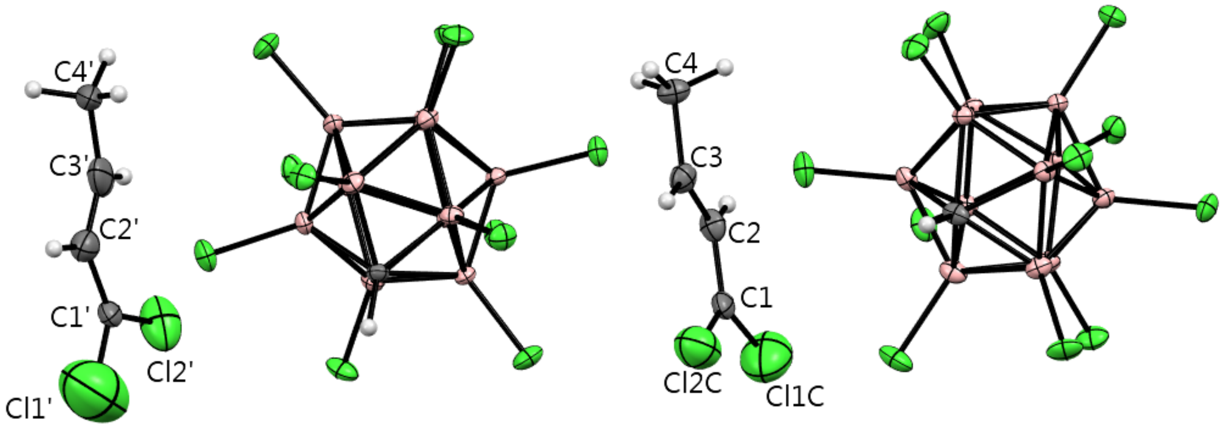

Figure S2. Crystallographically independent 1,1-dichlorobutylene cations and $\{Cl_{11}^{-}\}$ anions in the crystal lattice of their salt.

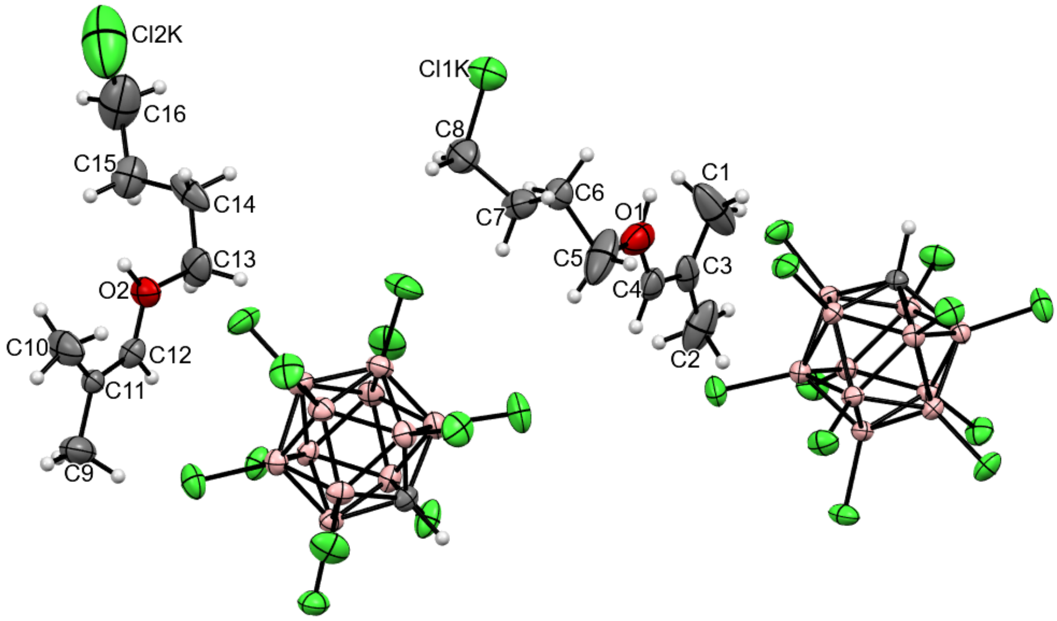

Figure S3. Crystallographically independent cations **I** and $\{Cl_{11}^{-}\}$ anions in the crystal lattice of their salt.

Crystal phase data

Table S1

X-ray data for compounds 1,1-dichloro-1-butylene carbocation and cations **I** and **II**

Compound	1,1-dichloro-1-butylene$^+$	**I**	**II**
Empirical formula	$C_4H_5Cl_2$+$CHB_{11}Cl_{11}$	$C_8H_{16}OCl$ + $CHB_{11}Cl_{11}$	$C_8H_{13}OCl_2$ + $CHB_{11}Cl_{11}$
Formula weight	645,86	685.54	717.96
Temperature K	200(2)	200(2)	200(2)
Wavelength Å	0.71073	0.71073	0.71073
Crystal system	Triclinic	Monoclinic	Monoclinic
Space group	P-1	$P2_1/c$	$P2_1/c$
Unit cell dimensions *a* Å	12.5834(5)	16.258(4)	16.355(5)
b Å	14.4684(8)	23.118(5)	9.979(3)
c Å	15.0626(8)	15.126(3)	17.462(5)
α °	104.414(2)	90	90
β °	110.117(2)	103.689(9)	96.166(11)
γ °	100.501(2)	90	90
Volume $Å^3$	2383.3(2)	5524(2)	2833.3(14)
Z	4	8	4
Density (calcd.) $Mg.m^{-3}$	1.760	1.649	1.683
Abs. coefficient mm^{-1}	1.391	1.210	1.275
F(000)	1240	2704	1408
Crystal size mm^3	0.04 x 0.15 x 0.30	0.20 x 0.30 x 0.30	0.005 x 0.01 x 0.30
Θ range for data collection °	1.52 – 26.09	1.29 - 30.26	2.35 – 25.03
Index ranges	$-15 \leq h \leq 15$, $-17 \leq k \leq 17$, $-18 \leq l \leq 18$	$-23 \leq h \leq 23$, $-32 \leq k \leq 32$, $-19 \leq l \leq 21$	$-19 \leq h \leq 19$, $-11 \leq k \leq 11$, $-20 \leq l \leq 20$
Reflections collected	25683	93831	24561
Independent reflections	9386 R(int) = 0.071	16365 R(int)= 0.056	4986 R(int)= 0.155
Completeness to θ %	99.7	99.7	99.6
Data / restraints / parameters	9386 / 0 / 531	16365 / 16 / 633	4986/42 / 308
Goodness-of-fit on F^2	1.03	1.05	1.12
Final R indices $I > 2\sigma(I)$	R_1 = 0.0627, wR_2 = 0.1659	R_1 = 0.0573, wR_2 = 0.1613	R_1 = 0.1180, wR_2 = 0.2667
Final R indices (all data)	R_1 = 0.1028, wR_2 = 0.1938	R_1 = 0.0722, wR_2 = 0.1733	R_1 = 0.2138, wR_2 = 0.2893
Largest diff. peak / hole $e.Å^{-3}$	1.28 / -1.25	1.57 / -1.30	0.96/ -0.78
CCDC			

Table S2. Selected bond lengths (Å) and bond angles (°) for the 1,1-dichloro-1-butylene cation (two independent molecules):

Molecule 1		Molecule 2	
Bond	Length (Å)	Bond	Length (Å)
C1–Cl1c	1.629(9)	C1’–Cl1c’	1.601(9)
C1–Cl2c	1.671(8)	C1’–Cl2c’	1.621(8)
C1–C2	1.395(10)	C1’–C2’	1.359(11)
C2–C3	1.325(11)	C2’–C3’	1.299(13)
C3–C4	1.497(10)	C3’–C4’	
Bond angles	(°)	Bond angles	(°)
Cl1c–C1–Cl2c	119.6(5)	Cl1c’–C1’–Cl2c’	117.5(6)
Cl1c–C1–C2	118.0(6)	Cl1c’–C1’–C2’	115.3(7)
Cl2c–C1–C2	122.2(6)	Cl2c’–C1’–C2’	126.9(6)
C1–C2–C3	121.8(7)	C1’–C2’–C3’	124.0(9)
C2–C3–C4	125.0(6)	C2’–C3’–C4’	129.7(10)

Table S3. Selected bond lengths (Å) and bond angles (°) for cation **I** (two independent molecules):

Molecule 1		Molecule 2	
Bond	Length (Å)	Bond	Length (Å)
Cl1K–C8	1.775(5)	Cl2K–C16	1.738(9)
O1–C4	1.269(5)	O2–C12	1.287(4)
O1–C5	1.435(9)	O2–C13	1.475(6)
O1–C5’	1.435(9)	O2–C13’	1.475(6)
C1–C3	1.451(9)	C9–C11	1.456(5)
C2–C3	1.455(8)	C10–C11	1.456(7)
C3–C4	1.274(6)	C11–C12	1.278(5)
C5–C6	1.565(9)	C13–C14	1.524(10)
C5’–C6’	1.50(2)	C13’–C14’	1.40(2)
C6–C7	1.518(9)	C14–C15	1.547(13)
C6’–C7’	1.52(3)	C14’–C15’	1.50(2)
C7–C8	1.528(8)	C15–C16	1.563(12)
C7’–C8’	1.50(2)	C15’–C16’	1.55(1)
Bond angles	(°)	Bond angles	(°)
C4–O1–C5	117.1(4)	C12–O2–C13	115.8(3)
C1–C3–C2	119.6(5)	C9–C11–C10	117.5(4)
C1–C3–C4	120.4(5)	C9–C11–C12	120.4(3)
C2–C3–C4	120.1(4)	C10–C11–C12	121.9(3)
O1–C4–C3	121.8(4)	O2–C12–C11	121.5(3)
O1–C5–C6	98.8(5)	O2–C13–C14	108.0(4)
O1–C5’–C6’	132(1)	O2–C13’–C14’	109.5(8)
C5–C6–C7	105.1(5)	C13–C14–C15	107.5(7)
C5’–C6’–C7’	109(2)	C13’–C14’–C15’	128(2)
C6–C7–C8	109.0(5)	C14–C15–C16	112.1(7)
C6’–C7’–C8’	111(2)	C14’–C15’–C16’	112(1)
Cl1K–C8–C7	114.2(4)	Cl2K–C16–C15	129.6(7)
Cl1K–C8’–C7’	113.2(9)	Cl2K–C16’–C15’	92.6(6)
